# Metformin use and early lactate levels in critically ill patients according to chronic and acute renal impairment

**DOI:** 10.1186/s13054-020-03300-y

**Published:** 2020-09-29

**Authors:** Rene A. Posma, Adam Hulman, Reimar W. Thomsen, Bente Jespersen, Maarten W. Nijsten, Christian F. Christiansen

**Affiliations:** 1 Department of Critical Care, University of Groningen, University Medical Center Groningen, Groningen, The Netherlands; 2 Department of Clinical Epidemiology, Aarhus University Hospital, Aarhus, Denmark; 3 Steno Diabetes Center Aarhus, Aarhus University Hospital, Aarhus, Denmark; 4 Department of Renal Medicine, Aarhus University Hospital, Aarhus, Denmark

**Keywords:** Metformin, Lactate, Acute kidney injury, Chronic kidney disease, Metformin-associated lactic acidosis, Critical care

## Main text

Metformin is the most widely used oral antihyperglycemic agent. Because it is eliminated unmodified in urine, patients with renal insufficiency can accumulate metformin and may develop lactic acidosis [1]. Recent guidelines only restrict the use of metformin in patients with severe chronic kidney disease (CKD) because the benefit is considered larger than the risk for lactic acidosis [2]. Lactate measurement has a central role in identifying and monitoring critical illness [3]. A better understanding of the impact of metformin on lactate levels could improve clinical assessment of the critically ill.

Data were collected by combining data from Danish nationwide medical databases with laboratory data [4]. This multicenter cohort included all adults (≥ 18 years) hospitalized and surviving 24 h of intensive care unit (ICU) treatment in northern Denmark between January 2010 and August 2017. We required ≥ 3 lactate measurements between 6 h before until 24 h after ICU admission, with ≥ 12 h between first and last measurement. Patients receiving dialysis before ICU admission were excluded.

Metformin use was defined as a filled prescription for metformin within 90 days before ICU admission [4]. CKD stage was assessed by the mean estimated glomerular filtration rate (eGFR) 365 days until 7 days before ICU admission [5]. Acute kidney injury (AKI) within 24 h after ICU admission was defined and staged according to the KDIGO creatinine criteria. Lactate trajectories over time for metformin users and nonusers were fitted by a mixed-effects model assuming unstructured covariance and including individual-level random intercept and slope. Time was modeled as a natural cubic spline with knot locations at − 1 h, + 4 h, and + 12 h relative to ICU admission. Time-by-group interaction was entered as a covariate, and analyses were subsequently stratified by eGFR level or AKI stage. Differences in maximum lactate level with 95% confidence intervals between metformin users and nonusers were model-based.

**Fig. 1:**
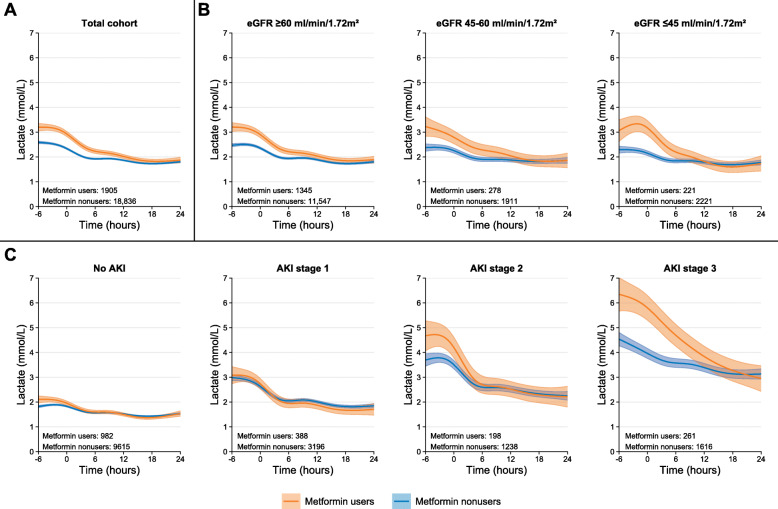
Lactate levels for metformin users and nonusers according to estimated glomerular filtration rate and acute kidney injury stage. Mean lactate trajectories over time with 95% confidence interval for metformin users and nonusers were fitted by a mixed-effect model with individual-level random intercept and slope. Time after ICU admission was modeled as natural cubic spline with knot location at − 1 h, + 4 h, and + 12 h surrounding intensive care unit admission. **a** Total population. Subsequently, analyses were stratified according to **b** chronic kidney disease stage based on mean estimated glomerular filtration rate (eGFR) 1 year before ICU admission or **c** acute kidney injury (AKI) stage within 24 h of ICU admission

We studied 20,741 patients with a total of 209,394 lactate measurements, of whom 1905 (9%) patients used metformin (Table 1). Compared with nonusers, metformin users had a similar preadmission eGFR but had more often AKI stage 2 or 3. Metformin users had 0.61 (0.45–0.77) mmol/L higher maximum lactate levels than nonusers (Fig. 1a). This difference was highest for patients with eGFR ≤ 45 ml/min/1.73 m^2^ (1.06 [0.72–1.39] mmol/L; Fig. 1b). Differences in maximum lactate levels between metformin users and nonusers were more pronounced in patients with AKI stage 2 or 3 (Fig. 1c), with a difference of 0.30 (0.15–0.45) mmol/L for patients without AKI, and 0.12 (− 0.24 to 0.48), 1.00 (0.35–1.65), and 1.75 (1.03–2.47) mmol/L among patients with AKI stage 1, 2, or 3, respectively. The difference between metformin users and nonusers disappeared within 24 h of ICU admission. However, the time until this difference disappeared was longer for patients with moderate to severe CKD or AKI (Fig. 1).

**Table 1:** Characteristics of metformin users and nonusers

Characteristic	Total (***N*** = 20,741)	Metformin users (***N*** = 1905)	Metformin nonusers (***N*** = 18,836)	SMD*
Age, median [IQR], years	69 [58–77]	70 [63–76]	69 [58–77]	0.24
Male sex	11,697 (56)	1193 (63)	10,504 (56)	0.14
Charlson Comorbidity Index				0.52
0	6894 (33)	305 (16)	6589 (35)	
1 or 2	8147 (39)	737 (39)	7410 (39)	
3 or higher	5700 (27)	863 (45)	4837 (26)	
Diabetes mellitus	4594 (22)	1903 (100)	2691 (14)	3.45
Sulfonylureas	476 (2)	250 (13)	226 (1)	0.48
Insulin	1473 (7)	443 (23)	1030 (5)	0.52
Other antihyperglycemic agents	445 (2)	259 (14)	186 (1)	0.50
Preadmission eGFR, median [IQR], ml/min/1.73 m^2^	80 [58–95]	77 [58–92]	80 [58–95]	0.07
≥ 60 ml/min/1.73 m^2^	12,892 (62)	1345 (71)	11,547 (61)	0.48
45–60 ml/min/1.73 m^2^	2189 (11)	278 (15)	1911 (10)	
≤ 45 ml/min/1.73 m^2^	2442 (12)	221 (12)	2221 (12)	
Missing	3218 (16)	61 (3)	3157 (17)	
ICU admission type				0.20
Medical	9942 (48)	1019 (53)	8923 (47)	
Emergency surgical	6344 (31)	456 (24)	5888 (31)	
Elective surgical	3149 (15)	345 (18)	2804 (15)	
Missing	1306 (6)	85 (4)	1221 (6)	
Time from hospital admission to ICU admission, median [IQR], h^†^	5.1 [0.0–30.5]	5.3 [0.0–29.4]	5.1 [0.0–30.7]	0.06
SAPS-II score, median [IQR]	40 [30–52]	42 [31–53]	40 [30–52]	0.09
Missing	11,456 (55)	1024 (54)	10,432 (55)	
Mechanical ventilation	9305 (45)	815 (43)	8490 (45)	0.05
Inotropes or vasopressors	8943 (43)	854 (45)	8089 (43)	0.04
Renal replacement therapy	1257 (6)	152 (8)	1105 (6)	0.09
AKI stage within 24 h				0.47
No AKI	10,597 (51)	982 (52)	9615 (51)	
1	3584 (17)	388 (20)	3196 (17)	
2	1436 (7)	198 (10)	1238 (7)	
3	1877 (9)	261 (14)	1616 (9)	
Missing	3247 (16)	76 (4)	3171 (17)	
30-day mortality	4367 (21)	346 (18)	4021 (21)	0.08

Data are expressed as no. (%) or median [IQR]

*As general guidance, it is suggested that effect sizes are likely to be “small” when an SMD approximates 0.2, likely to be “medium” when an SMD is 0.5, and “large” when an SMD is higher than 0.8

^†^In total, data are missing for 19 (0.1%) patients

*SMD* standardized mean difference, *eGFR* estimated glomerular filtration rate, *ICU* intensive care unit, *SAPS-II* Simple Acute Physiology Score II, *AKI* acute kidney injury

In this large cohort of critically ill patients, metformin users had higher lactate levels than nonusers in the early phase of critical illness, which disappeared within 24 h of ICU admission. Importantly, the difference in lactate levels between metformin users and nonusers was higher in patients with more severe AKI, while the difference was almost similar across preadmission eGFR subgroups. This may be explained by reduced clearance of metformin or lactate, or both. A limitation is that blood metformin concentrations were unavailable to confirm this because such correlation was found in patients receiving renal replacement therapy for metformin-associated lactic acidosis [6].

The monitoring of lactate trajectories is recommended during critical illness [3]. Awareness of factors affecting this biomarker will improve its interpretation. We report that the association of metformin use with increased lactate levels is more pronounced in patients who develop AKI stage 2 or 3 than in patients without AKI or who develop AKI stage 1.

## Data Availability

Parts of the data that support the findings of this study are available from the Danish Health Data Authority (Sundhedsdatastyrelsen), but restrictions apply to the availability of these data, which were used under license for the present study and are thus not publicly available.

## Abbreviations

CKD: Chronic kidney disease
ICU: Intensive care unit
eGFR: Estimated glomerular filtration rate
AKI: Acute kidney injury
KDIGO: Kidney Disease Improving Global Outcomes
SMD: Standardized mean difference
